# The effectiveness of emerging markets’ legal structure in explaining financial development

**DOI:** 10.1371/journal.pone.0299831

**Published:** 2024-04-18

**Authors:** Muhammad Khalid Anser, Muhammad Asif Khan, Mohammed Arshad Khan, Wang Huizhen, Ahsanuddin Haider

**Affiliations:** 1 School of Business, Xi’an International University, Xi’an, Shaanxi, P.R. China; 2 Department of Commerce, Faculty of Management Sciences, University of Kotli, AJK, Pakistan; 3 Department of Accountancy, College of Administrative and Financial Sciences, Saudi Electronic University, Riyadh, Saudi Arabia; 4 Institute of Free Trade Zone, Xi’an International University, Xi’an, Shaanxi, P.R. China; 5 Department of Finance, College of Administrative and Financial Sciences, Saudi Electronic University, Riyadh, Saudi Arabia; Cavendish University / Kyambogo University, UGANDA

## Abstract

This article examines the role of legal structure in explaining financial development in twenty-three emerging markets, which has not been explored in institutional economics literature before. This study relied on Pedroni, and Kao cointegration tests, which is followed by the renowned panel cointegration technique. The results of the Pedroni and Kao cointegration tests show that the variables understudy is cointegrated in the long-run. These findings are confirmed by the panel cointegration showing that legal structure (LS) has positive impact on financial development (FIND) in long-run that support Law and Finance, and New Institutional Economics theories in emerging markets. This study is the first to directly examine the long-run impact of LS on FIND in emerging markets, and the result remains consistent across alternative measure of FIND. The findings of this study have important policy implications for emerging markets. Policymakers should focus on creating a legal environment that is conducive to financial development. This includes strengthening the legal framework, improving regulatory regimes, and promoting market autonomy. Additionally, policymakers should work to attract foreign investment, which can help spur economic growth and development in emerging markets. The findings of the study are consistent across battery of robustness testing.

## 1. Background

**Fig 1:**
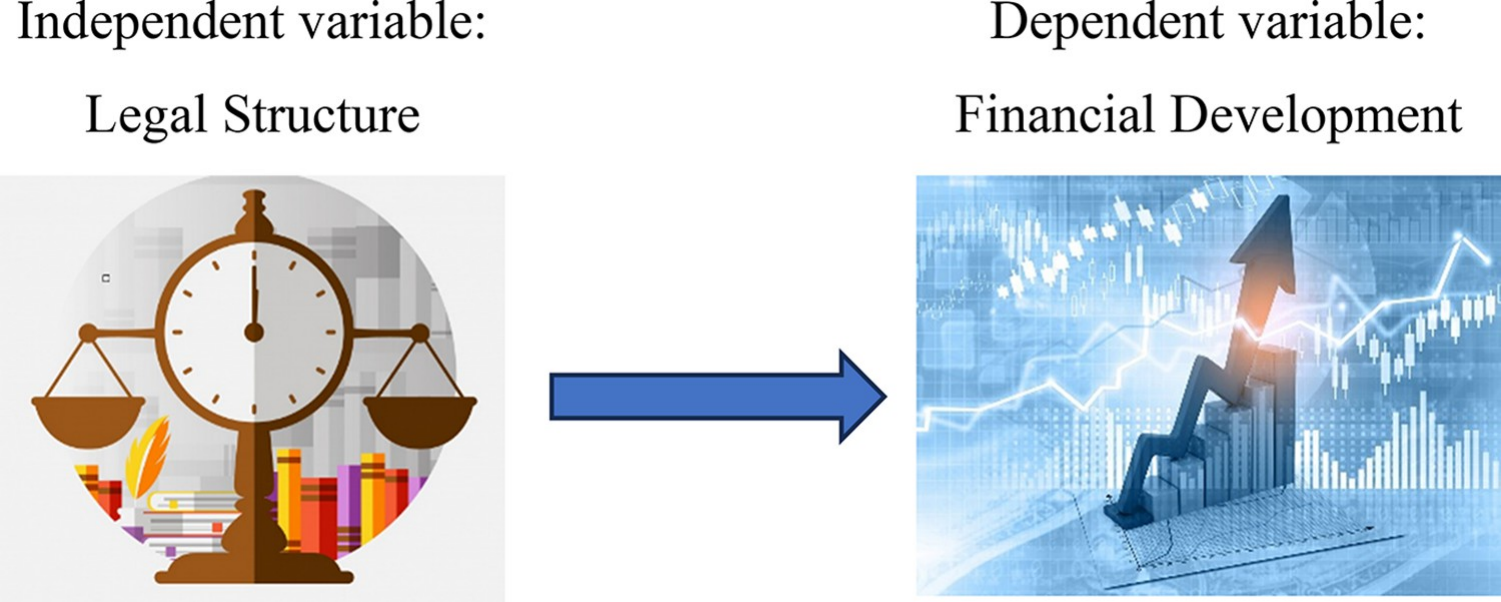
Illustrates the theoretical framework (drawn on: New Institutional Economics [4], and Law and Finance [5–7] Theories).

Institutional quality and financial development (FIND) are crucial pillars of economic development [1, 2] because they affect the allocation and productivity of resources, the incentives and opportunities for innovation and entrepreneurship, and the stability and resilience of the economy. Institutional quality affects economic development by influencing the costs and benefits of economic activities, the protection and enforcement of economic rights, and the credibility and accountability of economic policies [3]. While the FIND affects economic development by facilitating the mobilization and allocation of savings, the diversification and management of risks, and the transmission and implementation of monetary policies. The theoretical literature on institutions, finance, and economic development includes New Institutional Economics [4] and Law and Finance [5–7] provides base for understanding the underpinning role of institutional quality in boosting the economic development (specifically–FIND). North [4] emphasizes the role of institutions in reducing transaction costs, contract enforcement, and protection of property rights to promote economic growth. La Porta [5–7] argue that legal institutions can reduce the enforcement (contracts) and protection (property-rights) costs, making financial markets more attractive for investment. Institutional economics literature has established the link between institutional quality, FIND, and economic growth. Fig 1 shows that theoretical framework of the present study which is drawn on the New Institutional Economics [4] and Law and Finance [5–7].

On the empirical front, researchers have highlighted the significance of institutional quality and FIND as crucial contributing factor to economic development [1, 8–10]. Additionally, a body of scholarly work confirms that high-quality institutions play a vital role in promoting the development of the financial sector [2, 8, 10–14]. The empirical evidence demonstrates that institutions, both in their overall quality and specific dimensions, either facilitate or impede the financial sector development. Moreover, in certain cases, institutional quality is considered a moderating factor in finance and growth nexus [10, 11]. Therefore, understanding the factors that contribute to the formation of "good" institutions, are those which lead to contribute to the advancement of an economy’s financial sector, which is of paramount importance [11]. Therefore, it can be argued that institutions and financial development (markets, and institutions) serve as the fundamental pillars of emerging markets.

The literature on institutional impact on financial sector is extensively studied [1, 2, 12, 14–16]. However, an unexplored question pertains to how the effective legal structure (LS) mobilizes the FIND in emerging markets. Drawing upon the classical institutional finance theories [4, 6, 7], and recognizing the pivotal role of law in finance within emerging markets [1, 2, 12, 14–16], as well as the progressive significance of emerging markets on the global stage, it becomes imperative to investigate the impact of LS on FIND in an important group of emerging markets, which is unaddressed question in literature. To fill this gap, this is an effort to examine how effectiveness of LS influences the FIND in the cluster of twenty-three emerging markets based on Financial Times Exchange Group classification 2022.

This study empirically finds the evidence that LS has a positive impact on FIND (all its indicators studied) in emerging markets, which supports the theoretical frameworks of New Institutional Economics, and Law and Finance, suggesting that a well-established legal framework is indispensable for FIND in these markets. In addition, this study offers useful policy input for other stakeholders, such as investors, financial institutions, and international organizations. A well-established legal system in emerging markets can make foreign investors more willing to invest in financial markets, as it can lower the risk of expropriation and increase transparency. Financial institutions can also benefit from a stable legal environment, as it can enhance their ability to manage risk and promote innovation. Overall, these policy implications highlight the critical role that a well-established LS can play in promoting FIND and attracting capital flows across national borders, benefiting a range of stakeholders.

The empirical literature is reviewed in Literature Review section. The data, variables description, and econometric model are explained in Empirical Strategy section. The results are interpreted in the Empirical Results section. Discussion with reference to related studies is made in Discussion section, which is followed by Conclusion section.

## 2. Literature review

In developing countries, particularly those commonly referred to as "emerging markets," there has been a notable improvement in the ability to manage financial flows. In these markets the demand for financial services has considerably increased, which amplified the need for an efficient regulatory framework to deal with the sensitive nature of financial activities. Economic models that were previously employed in industrialized nations are now being applied in developing countries as well. The Mundell-Fleming model is a useful tool to understand how monetary and fiscal policies operate in emerging markets with high capital mobility [17]. Capital mobility refers to the ease of moving capital across national borders, which economists favor because it allows capital to seek the highest returns. Free capital movements have several advantages [18]. These include risk reduction through diversification of lending and investment, diffusion of best practices in corporate governance, accounting, and legal standards, and discipline of government policies by the global market. In a globally interconnected capital market with diverse capital flows into emerging markets, significant advancements are realized in capital markets.

Likewise, there is a widely held belief that a robust economy hinges on the presence of a entrenched rule of law, encompassing protections for individuals and their assets, constraints on government authority, and mechanisms to combat corruption [9, 19, 20]. However, certain conclusions about the sanctuary of private property may be deemed improbable due to the limited correlation between various rule of law components in developing nations. According to Johnson, McMillan [21], businesses are less likely to reinvest their profits if they do not have strong property rights, even if bank loans are accessible to them. Furthermore, businesses prefer to reinvest their profits in strong property right protection areas, rather than in the areas where such rights are feebler.

On one hand, the increasing influx of capital into emerging markets amplifies the pace of activities within capital markets. The trading of new multinational equity stock, tax-driven tech startups, and financial contracts for investment and lending has boosted the speed of global financial integration. However, this also poses challenges for regulators and institutions, who must cope with the increasing procedural requirements of securities and exchange commissions. Some of the challenges that arise from global financial integration are the licensing process, regulatory issues related to foreign firms’ equity stock registration, firms’ bank accounts, reporting and taxation necessities, religious, and cultural compliance, and security concerns. Therefore, emerging markets are needed to maintain encouraging legal structure to be comprehensive and conducive enough to support effective and efficient contract enforcement and protect the property rights of investors and lenders. Hayo and Voigt [22] argue that too much emphasis on procedural compliance may reduce the economic productivity potentially driven by corruption (particularly the rent seeking).

A well-defined and enforced legal system can provide a foreseeable and stable circumstances for financial market participants, which can lead to increased financial activities and resulting economic growth [23]. This, in turn, helps to attract investment, promote economic growth, and create jobs. legal structure (LS) is one of the key institutions that affects the efficiency and innovation of financial markets, as it determines the rules and regulations that govern financial contracts and instruments [24]. One important aspect of the LS of a country is the existence of laws that govern financial transactions. These laws may cover areas such as securities regulation, corporate governance, bankruptcy, and contract law [25]. When these laws are well-defined and enforced, investors and businesses are more likely to have confidence in the integrity of financial markets [26]. Another important aspect of a country’s LS is the independence of its judiciary. A strong and independent judiciary can ensure that disputes related to financial transactions are resolved fairly and efficiently–eventually helping the creation of a stable and predictable legal environment for financial market participants. The LS eases the flow of capital between investors and businesses, and they can also provide valuable services such as underwriting, market making, and research.

LS is one of the key institutions that affects the efficiency and innovation of financial markets, as it determines the rules and regulations that govern financial contracts and instruments [24]. Johnson, McMillan [21] believe that when property rights are weak, firms are discouraged from reinvesting their profits, even in the presence of available bank loans. In contrast, in regions with strong property rights, firms actively reinvest their profits. To attract investment and promote the growth of financial institutions, countries may need to create a regulatory environment that balances the needs of investors and businesses with the need to protect consumers and ensure financial stability. Overall, the LS of a country plays a valuable role in the expansion of the financial sector. Beck, Demirgüç‐Kunt [27] underscore the challenging nature of pinpointing the exact mechanism by which legal tradition exerts its influence on financial system. Nevertheless, it may affect FIND through various channels, including the regulatory framework, investor protection, contract enforcement, corporate governance, and judicial independence [4, 6, 7]. By concluding this section, we postulated that LS is important for boosting financial activities in emerging markets.

## 3. Empirical strategy

### 3.1 Data, and the variables description

To analyze the impact of LS on FIND in the sample of 23 FTSE classified emerging markets, the annual available secondary data for 1980–2022 is sourced from various sources. FIND is a dependent variable that measures the level of development and expansion of overall financial system in an economy–including depth, efficiency, and access of both financial institutions, and markets. It is an index ranging from 0 to 1, which is calculated based on the scores of various financial markets, and institutions indicators, such as depth, access, and efficiency (its composition and methodology is accessible in Svirydzenka [28]. The data is downloaded from the official website of IMF (IMF Strategy, Policy, and Review Department). This index is developed in Svirydzenka [28], and recently used in literature to proxy the FIND due to its wider coverage and comprehensive nature [16, 29–35].

The variable LS captures the effectiveness of a LS, which varies from 0 to 10. The index is composed of the following indicators: a) Judicial independence–The judiciary operates independently and is not influenced by the government or parties in dispute, and b) Impartial courts—A reliable legal framework exists for private businesses to challenge the legality of government actions or regulations, Protection of intellectual property, Military interference in rule of law and the political process, Integrity of the legal system. The variable LS measures the effectiveness of an LS on a scale from 0 to 10. A low score of 0 indicates the absence of structure, reliable legal framework, and intellectual property protection, while a high score of 10 indicates the existence of reliable lawful pursuits, and intellectual property shield. The data on LS is downloaded from the Quality of the Government (QOG) database which generated by Teorell, Sundström [36].

Among control variables, EG is the growth rate of real GDP per capita, which measures the change in the economic output of a country adjusted for population and inflation. EF is economic freedom, implying that how free are the citizens of a country to make decisions. Foreign direct investment, or FDI, is a type of cross-border investment that involves the transfer of various assets from foreign entities to domestic entities, involving resources, expertise, and capital. INV is the ratio of gross fixed capital formation to GDP, which measures the share of GDP that is invested in fixed assets such as machinery, equipment, and buildings. ER shows the value of one unit of a country in terms of USD. EF data is sourced from QOG database [36], while secondary data for all other control variables (EG, FDI, INV, ER) are downloaded from World Development Indicators of Word Bank [37].

### 3.2 Model specification

In this section, the rational of econometric model applied in this study is discussed with its essential pre-conditions.

#### 3.2.1 Panel unit root testing

We used panel data from 1980 to 2022 for twenty-three cross-sections, implying that time (T) is greater than cross sections (N). In present scenario T = 42 and N = 23, where T consists of longer time, and it is appropriate to test panel unit-root for the underlying variables before choosing suitable model. Khan, Popp [38] argue that in order to estimate the cointegration among the variables, we first need to find an integration order, as is usual for macro-level panels with long time series. Then, we choose appropriate panel data models based on the non-stationarity of the variables, as we do in this study. Thus, Im–Pesaran–Shin (IPS) panel unit root test is used to determine the integration order of the underlying variables. The IPS panel unit root test is a statistical method to test whether a panel of time series data contains a unit root, which means that the series is non-stationary and has a stochastic trend. The IPS test is based on the average of individual augmented Dickey-Fuller (ADF) tests for each panel, and it allows for heterogeneity in the autoregressive parameters and error variances across panels. The IPS test has more power than other panel unit root tests that assume a common autoregressive parameter for all panels, such as the Levin–Lin–Chu test. Hence our testing shows that none of the estimated variables are stationary at level, or second differences (the results are discussed in results section).

#### 3.2.2 Panel cointegration testing

After performing the IPS panel unit-root testing it is important to test panel cointegration. In this respect, the Pedroni [39] and Kao [40, 41] tests are two methods for examining the cointegration of panel data commonly used in macroeconomics, and finance. Cointegration means that there is a stable long-run relationship among the variables in the panel. The null hypothesis of both tests is that there is no cointegration. Under the null hypothesis, the test statistics follow a normal distribution as the number of panels and periods increases. The Pedroni test uses seven test statistics that are based on the residuals of the cointegrating regression. The test statistics can be classified into two groups: within-dimension and between-dimension. The within-dimension statistics pool the residuals across the cross-sectional units, while the between-dimension statistics average the individual unit root tests. The Pedroni test allows for heterogeneity in the slope coefficients and the error variances across the units. The Kao test uses a single test statistic that is based on the augmented Dickey-Fuller (ADF) regression. The Kao test assumes homogeneity in the slope coefficients and the error variances across the units. We proceed to test panel cointegration using Pedroni [39] and Kao [40, 41] panel cointegrations tests.

#### 3.2.3 Testing short, and long run relationship

The Pedroni and Kao cointegration tests only show whether there is a cointegration relationship among the panel data variables, but they do not provide the short-run and long-run estimates for each variable, nor the error correction term (ECT) that measures the speed of adjustment to the long-run equilibrium. Therefore, we use the Pooled Mean Group panel autoregressive distributed lag (PMG-PARDL) model to estimate the short-run and long-run coefficients of each variable in this study, and to determine the ECT. Eq (1) reflects the specification of generalized PARDL model.


$${FIND}_{it}=\sum_{j=1}^{p}{\delta_{ij}FIND}_{i,t-j}+\sum_{j=0}^{q}\beta_{ij}^{\prime }X_{i,t-j}+\varphi_{i}+e_{it}$$ (1)

In Eq (1), *FIND*_*it*_ is the dependent variable, (*X*_*i*,*t*_) is a k x 1 vector that are allowed to be purely I(0), or I(1) cointegrated; *δ*_*ij*_ is the coefficient of the lagged dependent variable called scalars; *β*_*ij*_ are k x 1 coefficient vector; *φ*_*i*_ is the unit-specific fixed effects; *i* = 1,…,*N*; *t* = 1,2,…,*T*; *p*, *q* are optimal lag orders; *e*_*it*_ is the error term. Eq (1) is transformed into error correction model in Eq (2).


$$\Delta {FIND}_{it}=\sum_{j=1}^{p-1}{\xi_{ij}\Delta FIND}_{i,t-j}+\sum_{j=0}^{q-1}\beta_{ij}^{\prime }{\Delta X}_{i,t-j}+{\rho_{i}FIND}_{i,t-1}+\lambda_{i}^{\prime }X_{i,t}+\varphi_{i}+e_{it}$$ (2)

In Eq (2) $\xi_{ij},\beta_{ij}^{\prime }$ are the short-run coefficients

***ρ***_***i***_ **=** group specific error correction coefficient

$\lambda_{i}^{\prime }$ = vector of long-run relationship

## 4. Empirical results

Table 1 shows the descriptive statistics of six variables: FIND, LS, EG, EF, FDI, and ER. Descriptive statistics are brief informational coefficients that summarize a given data set. The table reports the number of observations, standard deviation, mean value, minima, and maxima for each variable. The number of observations, or data points, in the data set is 989. The table shows that the mean of FIND is 0.2966, and for LS is 4.9777. The standard deviation of the variable, which measures how spread out the values are from the mean. A low standard deviation means that the values are close to the mean, and a high standard deviation means that the values are far from the mean. The table shows that the standard deviation of FIND is 0.1467, the standard deviation of LS is 1.2273. Similarly, the minimum and maximum values for each variable are also reflected in this table which ranges from 0 to 0.6923 for FIND, and 3.0045 to 8.9962 for LS.

**Table 1:** Descriptive statistics.

Variable	Obs	Mean	Std. Dev.	Min	Max
FIND	989	0.2966	0.1467	0	0.6923
LS	989	4.9777	1.2273	3.0045	8.9962
EG	989	14188.015	16807.997	162.6538	103264.86
EF	989	5.2893	1.0282	3.0057	7.4389
FDI	989	2.4002	5.3884	-39.9637	60.23906
INV	989	4.5085	9.9883	-47.9083	45.1193
ER	989	408.6435	1783.81	0.0814	16178.7823

Table 2 shows the pairwise correlations between five variables: LS, EG, EF, FDI, INV and ER. It measures the linear relationship between two variables using the Pearson correlation coefficient. The coefficient ranges from -1 to 1, where -1 indicates a perfect negative correlation, zero indicates no correlation, and one indicates a perfect positive correlation. The result shows that the highest correlation is between LS and EF (0.4711), which means that these two variables have a moderate positive relationship. The lowest correlation is between EG and ER (-0.1232), which means that these two variables have a weak negative relationship. The rest of the correlations are between -0.2 and 0.3, which indicate weak or negligible relationships.

**Table 2:** Pairwise correlations.

Variables	(1)	(2)	(3)	(4)	(5)	(6)
(1) LS	1.0000					
(2) EG	0.2520	1.0000				
(3) EF	0.4711	0.3542	1.0000			
(4) FDI	0.1802	-0.0120	0.2001	1.0000		
(5) INV	-0.0854	-0.1403	0.0345	0.0438	1.0000	
(6) ER	-0.1171	-0.1264	0.1039	-0.0094	0.0323	1.0000

Moreover, to assess the multicollinearity, we can also use the variance inflation factor (VIF), which is the inverse of the tolerance. Tolerance is the proportion of the variance of a variable that is not explained by the other variables in the model. A low tolerance (or a high VIF) indicates a high degree of multicollinearity. In panel data analysis, a common rule of thumb is that a VIF above 10 (or a tolerance below 0.1) indicates a serious multicollinearity problem. In Table 3, we can see that none of the VIF values are above 10, which means that there is no serious multicollinearity problem in the data.

**Table 3:** Variance inflation factor.

	VIF	1/VIF
EG	2.880	0.347
LS	2.780	0.360
EF	1.630	0.613
ER	1.240	0.807
FDI	1.070	0.937
INV	1.040	0.963
Mean VIF	1.700	-

Table 4 shows the results of the Im–Pesaran–Shin (IPS) test for unit roots in panel data. The IPS test is based on the augmented Dickey–Fuller (ADF) test for each panel, and then combines the individual test statistics into a group statistic. The null hypothesis of the IPS test is that all panels contain a unit root, and the alternative hypothesis is that some or all panels are stationary. The level t-statistics are compared with the critical values at the bottom of the table. If the level t-statistic is more negative than the critical value, then the null hypothesis of a unit root is rejected. If the level t-statistic is less negative than the critical value, then the null hypothesis of a unit root is not rejected. The table reports the t-statistics for the ADF test at the level and first difference of each variable. The results show that none of the level t-statistics are more negative than the critical values, so the null hypothesis of presence of a unit root is not rejected for any variable at the level. This means that all variables are non-stationary at the level, however, the t-statistics for the ADF test at the first difference of each variable compared with the critical values is more negative than the critical value. Which implies that all variables are stationary at the first difference.

**Table 4:** Panel unit root Im–Pesaran–Shin test results.

Variables	Level	First Difference	Remarks
FIND	-1.6832	-6.5554	Integrated at first difference
LS	-1.5934	-7.2818	Integrated at first difference
EG	-0.9598	-5.7776	Integrated at first difference
FDI	-1.8128	-7.6820	Integrated at first difference
INV	-1.7101	-4.7764	Integrated at first difference
EF	-1.3506	-7.2313	Integrated at first difference
ER	-1.7691	-6.1369	Integrated at first difference

Critical values: 1% (-1.930), 5% (-1.810), and 10% (-1.750)

### 4.1 Panel cointegration results

Table 5 shows the results of Pedroni and Kao panel cointegration tests. These statistics are used to test whether there is a long-run equilibrium relationship among non-stationary time series in a panel data setting. The null hypothesis of these tests is that there is no cointegration among the variables, and the alternative hypothesis is that there is cointegration. The table reports the test statistics and the p-values for each test type and each dependent variable: FIND and FIND_DC. If the absolute test statistic is higher than the critical value (or p-value is significant), then the null hypothesis is rejected. If the test statistic is less than the critical value (or p-value is insignificant), then the null hypothesis is not rejected.

**Table 5:** Panel cointegration test results.

Dependent variable	FIND		FIND_DC	
Test type:	Test statistics (p-value)	Remarks	Test statistics (p-value)	Remarks
**Pedroni test for cointegration:**				
Modified Phillips–Perron	-3.6664 (0.0001)	Cointegrated	-5.1649 (0.0000)	Cointegrated
Phillips–Perron	-2.9064 (0.0018)	Cointegrated	-1.4668 (0.0712)	Cointegrated
Augmented Dickey–Fulle	-5.3474 (0.0000)	Cointegrated	-2.0769 (0.0189)	Cointegrated
**Kao test for cointegration:**				Cointegrated
Modified Dickey–Fuller	-4.9879 (0.0000)	Cointegrated	-5.6339 (0.0000)	Cointegrated
Dickey–Fuller	-4.5343 (0.0000)	Cointegrated	-4.3975 (0.0000)	Cointegrated
Augmented Dickey–Fuller	-2.9010 (0.0019)	Cointegrated	-3.9560 (0.0000)	Cointegrated

The results show that all the test statistics are significant at various significance level, so the null hypothesis is rejected for all tests showing presence of cointegration relationship among the underlying variables (when FIND is taken as dependent variable). The remarks column summarizes the results by stating that the variables are cointegrated. For robustness, we replace the dependent variable with its near alternative measure domestic credit to private section as ration to GDP (FIND_DC) from World Development Indicators. The right part of the table shows that the test statistics for FIND_DC are also statistically significant, which implies that the cointegration relationship is holds for FIND_DC.

### 4.2 Long and short run estimation

Table 6 incorporates the results of the PMG-PARDL estimator (Eq 2) for a dynamic panel data model of the impact of LS on FIND. The PMG estimator allows the intercepts, short-run coefficients, and error variances to differ freely across groups, but constrains the long-run coefficients to be the same. The table reports the long-run and short-run coefficients of six independent variables: LS, EG, INV, EF, FDI, and ER, on the dependent variable FIND. The long-run coefficients indicate the cumulative or asymptotic effect of the LS on FIND, after considering the lagged effects of the variables. The table shows that all the long-run coefficients are statistically significant at the 1% level, except for ER, which is not significant. This means that LS, EG, INV, EF, and FDI have a significant and stable impact on FIND eventually, while ER does not. LS has a positive long-run impact on FIND as shown by coefficient of 0.0851, which means that a one-unit increase in LS leads to a 0.0851 unit increase in FIND, holding other variables constant, in the long run. EG has a negative coefficient of -0.3817, which means that a one-unit increase in EG leads to a 0.3817 unit decrease in FIND. This implies that EG has a negative effect on FIND in the long run, which could be due to crowding out or diminishing returns effects. INV has a positive and small, long-run coefficient of 0.0035, which means that a one-unit increase in INV, which is a measure of investment, leads to a 0.0035-unit increase in FIND in the long run. This implies that investment has a positive but weak effect on FIND in the long run, which could be due to financial deepening or capital accumulation effects. EF has a coefficient of 0.0638 which shows a positive long-run effect on FIND, which means that a one-unit increase in EF, which is a measure of EF, leads to a 0.0638 unit increase in FIND in the long run. FDI has a positive and exceedingly small, long-run coefficient of 0.0008, which means that a one-unit increase in FDI brings a 0.0008 unit increase in FIND, in the long run. This implies that FDI investment has a positive but negligible effect on FIND in the long run, which could be due to technology transfer or competition effects. However, the effect of ER is not statistically significant in the present case.

**Table 6:** Long-run, and short-run estimation Pooled mean group results.

	(1)
Variable	FIND
**Long run estimation:**	
LS	0.0851***
	(0.0189)
EG	-0.3817**
	(0.1693)
INV	0.0035**
	(0.0016)
EF	0.0638***
	(0.0109)
FDI	0.0008***
	(0.0000)
ER	0.00125
	(0.0015)
**Short run estimation:**	
ECT_t-1_	-0.1456***
	(0.0309)
LS _t-1_	-0.0066
	(0.0068)
EG _t-1_	-0.0296
	(0.0402)
INV _t-1_	-0.0002
	(0.0002)
EF _t-1_	-0.0134***
	(0.0048)
FDI _t-1_	-0.0076*
	(0.0044)
ER _t-1_	0.0014
	(0.0013)
Constant	0.0606***
	(0.0142)
Observations	504

Standard errors are in parentheses

*** p < .01

** p < .05

* p < .1

The short-run coefficients show the instantaneous or contemporaneous effect of the LS on FIND, holding other variables constant, in the same period. The table shows that only some of the short-run coefficients are statistically significant, and the signs and magnitudes of the coefficients vary across variables. This means that these variables have different and unstable impacts on FIND in the short run. Among these, EF and FDI negatively affect FIND in the short run. EF has a negative short-run coefficient of -0.0134, which is statistically significant at the 1% level. This means that a one-unit increase in EF leads to a 0.0134 unit decrease in FIND in the short run, holding other variables constant. This implies that EF has a negative and weak effect on FIND in the short run, which could be due to regulatory changes or transition costs effects. Likewise, FDI has a negative and small, short-run coefficient of -0.0076, which is statistically significant at the 10% level. This means that a one-unit increase in FDI leads to a 0.0076 unit decrease in FIND in the short run, holding other variables constant. This implies that foreign direct investment has a negative and weak effect on financial development in the short run, which could be due to capital outflows or displacement effects. The table also reports the error correction term (ECT), which measures the deviation of FIND from its long-run equilibrium level in the earlier period. The ECT has a negative sign and a value between 0 and 1, which shows the speed and direction of the adjustment. The ECT is -0.1456, which is statistically significant at the 1% level. This means that 14.56% of the deviation of FIND from its long-run equilibrium level is corrected in each period. This implies that the adjustment process is relatively slow, and it takes about seven periods for FIND to reach its long-run equilibrium level after a shock.

### 4.3 Robustness: Alternative measure of dependent variable

The PMG regression analysis in Table 7 examines the effect of LS on FIND using a different indicator of FIND, namely FIND_DC (the ratio of domestic credit to private sector to GDP). The table presents the long run and short run estimates of the explanatory variables, along with their standard errors, the ECT, and the intercept. The long run results show that LS, INV, and EF have a positive relationship with FIND_DC, while EG and ER have a negative one. The short run results reveal that INV and EF have a negative impact on FIND_DC. The ECT coefficient is negative and statistically significant, implying a convergence to the long run equilibrium at an annual speed of 12.87%. These findings are in line with those in Table 6.

**Table 7:** Impact of LS on FIND. Robustness with alternative measure of FIND.

	(1)
	FIND_DC
**Long run estimation:**	
LS	0.1158***
	(0.0246)
EG	-8.0244**
	(3.3454)
INV	0.0488***
	(0.0189)
EF	0.4382***
	(0.1207)
FDI	0.0011
	(0.0010)
ER	-0.0003**
	(0.0001)
**Short run estimation:**	
ECT_t-1_	-0.1287**
	(0.0122)
LS _t-1_	0.0143
	(0.035)
EG _t-1_	0.0639
	(0.0446)
INV _t-1_	-0.0012***
	(0.0004)
EF _t-1_	-0.042***
	(0.0146)
FDI _t-1_	-0.0014
	(0.0012)
ER _t-1_	-0.0371
	(0.0229)
Constant	0.1789***
	(0.0503)
Observations	504

Standard errors are in parentheses

*** p < .01

** p < .05

* p < .1

### 4.4 Robustness with sub-dimensions of FIND

We dig further to evaluate the impact of LS on sub-dimensions of FIND. In this context the dependent variables are FIND_FI (financial institutions depth) and FIND_FM (financial markets depth), respectively. Table 8 shows the results of two PMG regression models, where coefficients stand for the long run and short run effects of the independent variables on the dependent variables. In the long run, LS has a positive and significant effect on both FIND_FI and FIND_FM, meaning that a better legal system is associated with higher levels of financial development in both dimensions. EG has a positive effect on FIND_FI and a negative effect on FIND_FM, but neither of them is statistically significant, implying that economic growth does not have a clear or consistent impact on FIND. INV has a positive and significant effect on FIND_FI and a positive but insignificant effect on FIND_FM, suggesting that higher investment is related to higher financial institutions depth, but not necessarily to higher financial markets depth. EF has a positive and significant effect on both FIND_FI and FIND_FM, indicating that greater EF is conducive to FIND in both dimensions. FDI has a positive but insignificant effect on both FIND_FI and FIND_FM, implying that foreign direct investment does not have a strong or reliable influence on financial development. ER has a positive and significant effect on FIND_FM and a positive but insignificant effect on FIND_FI, implying that a higher exchange rate is associated with higher financial markets depth, but not necessarily with higher financial institutions depth.

**Table 8:** Robustness with sub-dimensions of FIND.

	(1)	(2)
	FIND_FI	FIND_FM
**Long run estimation:**		
LS	0.0569***	0.1012***
	(0.0214)	(0.0203)
EG	0.058	-0.097
	(0.1003)	(0.3175)
INV	0.0036***	0.0006
	(0.0012)	(0.0016)
EF	0.0867***	0.0839***
	(0.0123)	(0.0128)
FDI	0.0011	0.0012
	(0.0010)	(0.0001)
ER	0.0001	0.0001***
	(0.0000)	(0.0000)
**Long run estimation:**		
ECT_t-1_	-0.1481***	-0.1403***
	(0.0212)	(0.0252)
LS _t-1_	0.003	-0.0121
	(0.0058)	(0.0151)
EG _t-1_	-0.0268	-0.0669**
	(0.069)	(0.0313)
INV _t-1_	-0.0002*	-0.0001
	(0.0001)	(0.0002)
EF _t-1_	-0.0324***	0.0058
	(0.0101)	(0.0128)
FDI _t-1_	-0.0014	-0.0012
	(0.0012)	(0.0012)
ER _t-1_	-0.0015	-0.0088
	(0.001)	(0.0062)
Constant	0.0242***	0.0976***
	(0.0057)	(0.0195)
Observations	504	504

Standard errors are in parentheses

*** p < .01

** p < .05

* p < .1

ECT has a negative and significant effect on both FIND_FI and FIND_FM, meaning that there is a tendency for convergence to the long run equilibrium at an annual rate of 14.81% for FIND_FI and 14.03% for FIND_FM. This also affirms the presence of a co-integration relationship among the variables. However, in the short run, the effects of the independent variables are generally weaker and less consistent than in the long run, except for EF, which has a negative and significant effect on FIND_FI and a positive and significant effect on FIND_FM, suggesting that economic freedom has opposite short run and long run impacts on FIND.

### 4.5 Robustness with alternative estimators (FMOLS and DOLS)

For robustness with alternative estimator, we use Panel Fully Modified Least Squares (FMOLS) and Panel Dynamic Least Squares (DOLS) to estimate the long-run impact of LS on FD. FMOLS and DOLS are two methods for estimating the long-run relationship between cointegrated variables in panel data. They both correct for the endogeneity and serial correlation of the regressors and produce unbiased and normally distributed estimators. FMOLS modifies the OLS estimator by subtracting the nuisance parameters, while DOLS augments the cointegrating regression with leads and lags of the first differences of the regressors. They have different advantages and disadvantages in terms of computational simplicity, small sample properties, and robustness to different specifications.

Table 9 presents four models. Models 1 and 2 show the cointegration impact of LS on FD using FMOLS and DOLS, respectively. Both models confirm that LS significantly affects FD in emerging markets in the long run, but with a slightly lower magnitude than the main model. We also estimate the long-run impact of LS on FD_DC, an alternative traditional measure of FD, and report the results in models 3 and 4. These models also support the long-run impact of LS on FD_DC. The long-run variance tests confirm the existence of cointegration relationships across all models. Therefore, Table 9 validates the main findings and robustness.

**Table 9:** Robustness with alternative estimators FMOL, and DOLS.

	(1)	(2)	(3)	(4)
	FMOLS	DOLS	FMOLS	DOLS
Variable	FD		FD_BM	
LS	0.0373**	0.0242**	0.0356**	0.0526**
	(0.0175)	(0.0113)	(0.0175)	(0.0240)
EG	-0.2042***	-0.3435***	-0.3510***	-0.4308
	(0.0482)	(0.1054)	(0.0482)	(0.3268)
INV	-0.1817***	0.0003	-0.0895*	-0.0028
	(0.0516)	(0.0011)	(0.0516)	(0.0022)
EF	0.1709***	0.1073***	0.0978***	0.0882***
	(0.0144)	(0.0083)	(0.0144)	(0.0163)
FDI	-0.0893**	-0.0705***	0.0693*	0.0033
	(0.0353)	(0.0213)	(0.0353)	(0.0030)
ER	0.0020	0.0020**	0.0053	0.0021**
	(0.0101)	(0.0011)	(0.0101)	(0.0010)
R-squared	0.5205	0.5351	0.5481	0.5019
Long-run variance	0.0150	0.0102	0.0361	0.0208

### 4.6 Further analysis to correct cross-sectional dependence

In this section we dig deeper into the analysis to deal with cross sectional dependence which is a widespread problem with panel data estimation. After executing panel fixed effect model, we test for cross-sectional dependence and Psarian’s test of cross-sectional independence statistics 8.730 against p-value of 0.0000 the null hypothesis of cross-sectional independence is rejected. It implies that there is existence of cross-sectional dependence in the sample. To deal with this issue we used Driscoll-Kraay (DK) robust standard error. DK is a nonparametric method that corrects the OLS estimator by subtracting the nuisance parameters that depend on the short-run dynamics and the covariance of the regressors and the errors. It is consistent under general forms of cross-sectional and serial correlation, but it requires a considerable time dimension and may have poor finite sample properties. The results of DK are reported in Table 10. The reported results show the significant positive effect of LS on FD across the sample which are consistent with those reported in baseline model.

**Table 10:** Additional analysis results.

	(1)
Variable	FD
	DK
LS	0.0245***
	(0.0064)
EG	0.1683***
	(0.0272)
EF	0.0824***
	(0.0075)
FDI	0.0007
	(0.0006)
ER	0.0174***
	(0.0022)
Constant	0.3078***
	(0.0593)
Observations	989
R-squared	0.4589

Standard errors are in parentheses

*** p < .01

** p < .05

* p < .1

## 5. Discussion

As demonstrated in this study, LS is a long-run determinant of FIND in emerging markets, and in short-run it does not have statistically significant impact on it. There are different theories and perspectives on the reasons for the relationship between LS and FIND in the long run and the short run. One possible explanation is that LS affects the quality and efficiency of financial contracts, markets, and intermediaries, which in turn influences the allocation of capital, the mobilization of savings, and the enforcement of property rights [23]. It validates the New Institutional Economics theory [4] and Law and Finance theory [5–7].

Similarly, Li [42] and Blundell, Dixit [43] have argued that the importance of formal institutions tends to increase as financial activities become more extensive. This is because formal institutions have high fixed costs and low marginal costs, which make them more efficient and effective in regulating financial markets in the long run. These factors have a cumulative and lasting impact on FIND in the long run, but may not be immediately noticeable or significant in the short run, especially when there are other factors that affect financial development, such as monetary policy, financial inclusion, and money laundering [44]. Another possible explanation is that legal structure is a slow-changing and path-dependent institution, which reflects the historical, political, and cultural factors of a country [23]. Therefore, legal reforms that aim to improve FIND may take a long time to be implemented, enforced, and accepted by society, and may face resistance or adaptation from the existing financial system. Thus, legal structure may not have a strong or consistent effect on FIND in the short run but may have a profound and persistent effect in the long run. Higher level of institutional quality incentivize favorable conduct among economic agents, and these institutions can take the form of either formal arrangements, such as legal enforcement of laws by the third-parties, such as self-enforceable contracts [45]. Hence, the entrepreneurial activities are boosted by the stronger LS [46].

A resilient and independent judiciary is a fundamental requirement for the protection of property rights, a critical element in driving financial market development, as underscored by Rodrik [45]. When investors have confidence that their investments are secure (protection of their property rights), they would be motivated to make investment in the financial market securities which is expected to increase the velocity of economic and financial activities. In line with Johnson, McMillan [21], businesses exhibit a reduced inclination to plough back the retained earnings in areas where their rights are compromised, even when access to bank loans is available. Instead, they tend to reinvest their profits in areas where property rights are well-established. Consequently, the legal framework plays a pivotal role in ensuring effective contract enforcement, particularly crucial in emerging economies marked by frail legal structures and inconsistent contract adherence. An impartial judiciary streamlines contract enforcement, establishing a stable and predictable legal milieu, serving as a prerequisite for the development of financial markets.

Literature establishes that corruption poses a substantial hindrance in the way of financial development in emerging markets [9, 47–49], that. An effective and autonomous LS boost (reducing corruption) confidence of financial market’s participants to attract FDIs when rule of law is upheld. This creates a level playing field for market participants and aids in attracting foreign investment, a vital part for financial market development. As documented in La Porta, Lopez-De-Silanes [6], that financial market’s transparency can be enhanced through sound legal enforcements. Consequently, autonomous judiciary will be in a better position to indulge in impartial decision-making (without political influence), thereby mitigate various forms of market-manipulation, and insider-trading risk. Eventually, this enhances the financial markets’ integrity and stability where investors feel confident in impartial courts which will result in lower uncertainties, and litigation costs as a result financial activities will excel [19, 50].

On the contrary Johnson, McMillan [21], firmly believe that weak intuitions have a detrimental impact on economic efficacy. Because those emerging markets often face a deficiency in the LS, which can be attributed to a range of deteriorating factors such as elevated level of corruption, political intervention, fewer competent judges, least financial resources, and weak legal frameworks. These multifaceted elements engender an environment of uncertainty, undermining investor confidence and subsequently exerting a negative influence on financial markets. The recent study conducted by Zhao and Zhang [51] corroborates the conclusions drawn in the present research, who demonstrate that enhancements in the LS positively influences the corporate finance, and financial system. This leads to a reduction in bribery expenditures, and increased investments in productive ventures, thereby fostering concurrent advancements in both the economy and the legal arrangement.

To conclude this section, we assert that the LS plays a pivotal role in shaping the financial development in emerging economies. It serves as a safeguard for property rights, eases contract enforcement, mitigates corruption, promotes transparency, and stimulates healthy competition, all of which are indispensable for the robust functioning of financial markets. These findings are in alignment with theoretical tenets such as New Institutional Economics and Law and Finance and are further substantiated by empirical research.

## 6. Conclusion

This study investigates the effect of legal structure on financial development in twenty-three emerging markets from 1980 to 2022 using panel data analysis. First, we conducted a panel unit-root test and confirmed that the variables are first-difference stationary. Then, we applied the Pedroni and Kao panel cointegration tests and found evidence of cointegration among the variables. Next, we used the PMG-PARDL technique to estimate the short-run and long-run relationships between legal structure and financial development, as well as the error correction term that measures the speed of adjustment to the long-run equilibrium. The results showed that legal structure has a significant and positive impact on financial development in the long run, but not in the short run, in these markets. Specifically, the ECT coefficients were -0.1456% (Table 6) and -0.1287% (Table 7), showing that the system converges to the long-run equilibrium at an annual rate of 14.56% and 12.87%, respectively. Furthermore, we employed FMOLS and DOLS to verify long-term dynamics and consistently observed the influence of legal structure on financial development. Additionally, addressing cross-sectional dependence using Driscoll-Kraay, the results were aligned with those obtained by baseline models. These findings support the theoretical frameworks of New Institutional Economics, and Law and Finance–highlighting the importance of a well-established legal framework for promoting financial activities.

Policymakers in emerging markets can use these findings to prioritize improving their legal systems as an effective tool for promoting financial and economic activities and resulting overall economic progress. In addition, the financial market participants can benefit from a legal system that is well-developed, as it creates an amicable environment for them. It lowers the costs of transactions, ensures the enforcement of contracts, and safeguards the rights of property owners. This is essential for the development of robust and efficient financial markets that can attract capital flows across national borders.

The main goal of this study was to explore how the legal system affects the financial sector development over a long period of time, assuming a linear relationship between the two variables. However, it is possible that the legal system may have a more complex and nonlinear influence on the financial sector, which could be an interesting avenue for further research (particularly, the threshold effect). Moreover, this study departs from fully testing the channels through which legal structure drives the financial development, and it is suggested that in future said channels may be explored in greater depth to provide a more comprehensive understanding of the implications of legal structure on financial development in emerging markets. Finally, this study does have limitations stemming from its reliance on macro variables and data. While these provide a broad overview of the impact of legal structure on financial development and its major dimensions, a more comprehensive understanding would necessitate exploring each sample economy individually.

## Supporting information

S1 File (ZIP)
